# Differential Metabolic Profiles during the Developmental Stages of Plant-Parasitic Nematode *Meloidogyne incognita*

**DOI:** 10.3390/ijms18071351

**Published:** 2017-06-24

**Authors:** Parthiban Subramanian, Byung-Ju Oh, Vimalraj Mani, Jae Kook Lee, Chang-Muk Lee, Joon-Soo Sim, Ja Choon Koo, Bum-Soo Hahn

**Affiliations:** 1 Department of Agricultural Biotechnology, National Institute of Agricultural Sciences, Rural Development Administration, Jeonju 54874, Korea; parthi@chungbuk.ac.kr (P.S.); byungjuoh@gmail.com (B.-J.O.); vimal@jbnu.ac.kr (V.M.); changmuk@korea.kr (C.-M.L.); jssim@korea.kr (J.-S.S.); 2 Division of Science Education and Institute of Fusion Science, Chonbuk National University, Jeonju 761-756, Korea; jkoo@jbnu.ac.kr; 3 Department of Agro-Food Safety and Crop Protection, National Institute of Agricultural Sciences, Rural Development Administration, Jeonju 54874, Korea; jk2lee@korea.kr

**Keywords:** root-knot nematode, *Meloidogyne incognita*, developmental stages, metabolic profiles, metabolic pathways

## Abstract

*Meloidogyne incognita* is a common root-knot nematode with a wide range of plant hosts. We aimed to study the metabolites produced at each stage of the nematode life cycle to understand its development. Metabolites of *Meloidogyne incognita* were extracted at egg, J2, J3, J4, and female stages and 110 metabolites with available standards were quantified using CE-TOF/MS. Analyses indicated abundance of stage-specific metabolites with the exception of J3 and J4 stages which shared similar metabolic profiles. The egg stage showed increased abundance in glycolysis and energy metabolism related metabolites while the J2 metabolites are associated with tissue formation, motility, and neurotransmission. The J3 and J4 stages indicated amino acid metabolism and urea cycle- related metabolites. The female stage was characterized with polyamine synthesis, antioxidant activity, and synthesis of reproduction related metabolites. Such metabolic profiling helps us understand the dynamic physiological changes related to each developmental stage of the root-knot nematode life cycle.

## 1. Introduction

*Meloidogyne incognita* is a soil pest causing major agricultural losses in crop plants [1,2]. With a host range of over 100 plants, this plant-parasitic nematode is a serious threat to cotton, tobacco, food legumes, vegetable crops, yams, potatoes, spices, and coffee. It is spread all over the world and has been reported in Asia, Africa, North, Central and South America, the Caribbean, Europe, and Oceania [3]. Different approaches to control such plant-parasitic nematodes (PPNs) are arising as hitherto employed methods are either detrimental to the environment or becoming obsolete [4,5,6]. The life cycle of this plant-parasitic nematode consists of five stages including egg, juvenile J2, J3, J4, and female/male. The eggs laid by female worms residing in the plant roots, develop into infective J2 juveniles. After infecting the host roots, the J2 worms develop feeding sites on the roots and turn sedentary. The third (J3) and fourth (J4) stage juveniles are sedentary in nature which molt into male and female adults. As male adults do not play a part in reproduction, female worms are important in the life cycle as they produce eggs which hatch to release J2 that continues to infect other roots. This diversity in morphology, localization, and function of nematode stages lead to the hypothesis that each developmental stage may have variable metabolic profiles.

After the report on the whole genome sequence of *M. incognita* in the year 2008, research has focused on a search for stage-specific genomic or molecular markers which can be developed into potential targets to control this plant-parasitic nematode [1,7]. Currently, there is no available resource on general and/or stage-specific metabolites from *M. incognita*. Earlier metabolic studies on nematodes have often dealt with the dauer stage metabolism in *Caenorhabditis elegans* or metabolites from infected plant roots [8,9,10]. These reports however were not able to provide a clear picture on the metabolism of plant-parasitic nematodes and *M. incognita* in particular as the nematode does not show a prominent dauer stage. In addition, samples from infected plant roots also contain plant metabolites, hindering a clear picture. Therefore, removing the nematodes from their hosts and studying metabolites present at various individual stages of the root-knot nematode life cycle can be a useful resource for improving our existing knowledge of the metabolism of nematodes and provide useful insights into their parasitism [11]. Unlike plants or microbes, secondary metabolite synthesis pathways do not commonly occur in animals and primary metabolism remains to be the fundamental source of biomolecules [12]. Stage-wise metabolic analyses in an earlier study indicated high variability in stage-specific metabolism among nematodes [11]. For example, the metabolic clusters expressed in early stage in *C. elegans* were not expressed in early stages of *Brugia malayi* [11]. This necessitates stage-wise metabolic studies in the nematode for a comprehensive picture on its metabolism. Therefore, in the present study, we collected the nematode *M. incognita* at five stages (egg, J2, J3, J4, and female) of its life cycle and profiled the metabolites produced.

## 2. Results and Discussion

Overall, the metabolites produced by the nematode were found to be shared among several metabolic pathways occurring in the cells. The metabolites and their related metabolism are given in Table 1. Among the observed metabolic pathways, the key pathways included amino acid metabolism, branched chain and aromatic amino acids metabolism, central carbon metabolism, lipid metabolism, metabolism of coenzymes, nucleotide metabolism, urea cycle related metabolism, and other metabolites which could not be grouped to any of the above metabolisms or were transiently formed.

**Figure 1:**
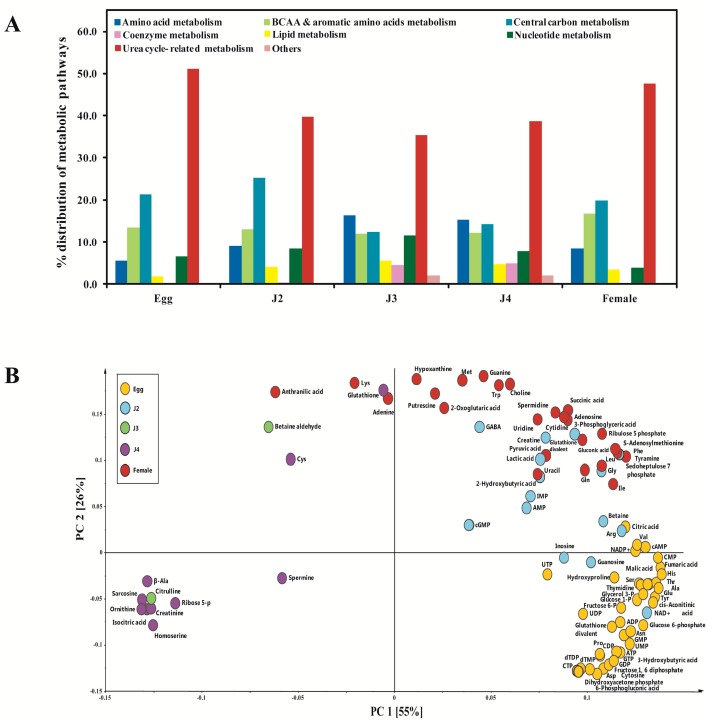
(**A**) Diversity of the metabolites observed at each stage of the nematode life cycle indicating major metabolic pathways of the cellular metabolism to which they constitute; (**B**) PCA loading plot of the two first principal components of the metabolites of *M. incognita* at various stages. The sum of two components amounted to 77.8%. Here, the metabolites can be observed to be grouped based on the stages where their expression has been significantly observed. The colored dots indicate the stage at which the particular metabolite showed highest quantification.

We were able to detect 93 metabolites among the 110 studied metabolites of which 67 were commonly synthesized at all stages (Table 2). These 93 metabolites constituted the above-mentioned metabolisms. Their levels of expression at each stage constituting to the overall metabolism were calculated. We found urea cycle to be the dominant metabolism at all stages of the nematode life cycle (Figure 1A). Excluding it, we found central carbon metabolism to be the major pathway in egg, J2, and female stages; whereas in the J3 and J4 stages, amino acid metabolism was the major pathway (Figure 1A). Principal component analysis (PCA) showed a clear co-relation between the stages and the metabolites synthesized (Figure 1B). Metabolites that were highly synthesized in their respective stages were found to align together, forming distinctive groups in the scatter plot (Figure 1B).

Study of stage-dependent regulation of metabolites based on their expression levels also indicated a trend similar mentioned as above (Table 3). For example, we observed Asp and 3-hydroxybutyric acid; ATP (adenosine triphosphate) and NAD^+^ (nicotinamide adenine dinucleotide) which constitute the central carbon metabolism to be upregulated in egg and J2 stages, respectively (Table 3).

**Figure 2:**
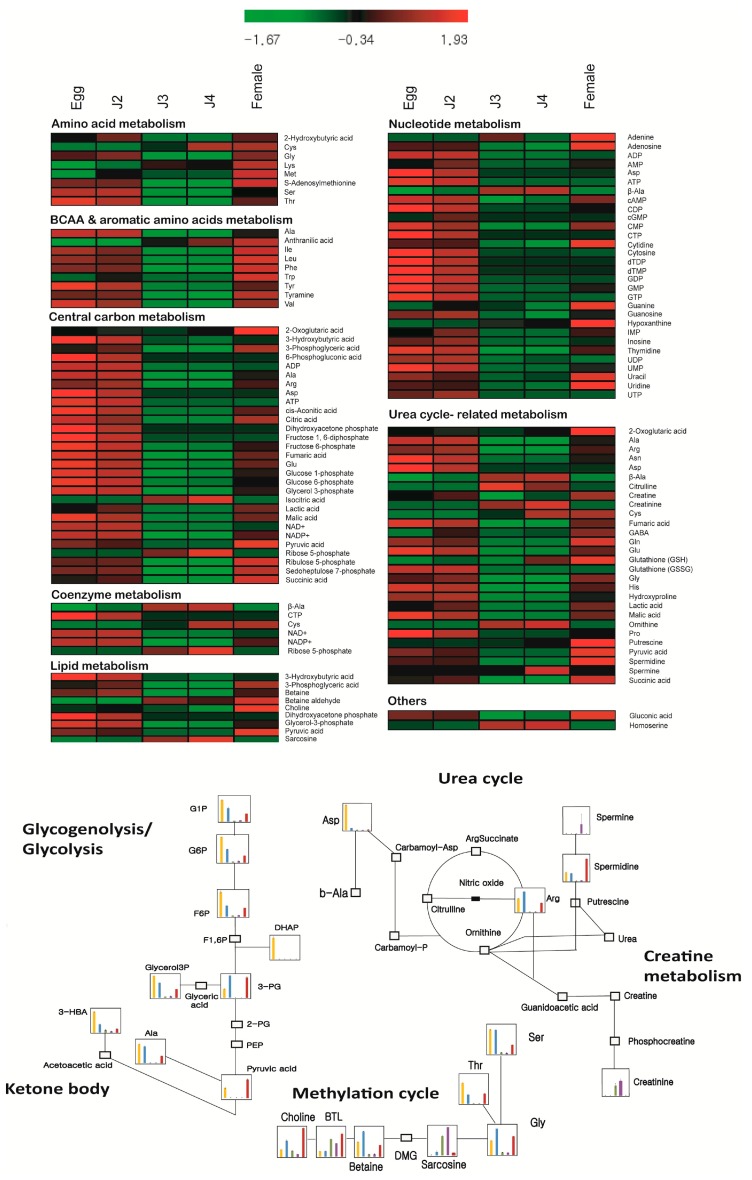
Heat map comparing the levels of 93 metabolites detected in *Meloidogyne incognita* at various stages of its life cycle. For construction of a comparative heatmap absolute quantified values were normalized and relative expression represented at a range of −1.67 to 1.93 was used. The metabolites were grouped based on their metabolism into amino acid metabolism, BCAA and aromatic amino acid metabolism, central carbon metabolism, coenzyme metabolism, lipid metabolism, nucleotide metabolism, urea cycle-related metabolism and others. Generation of the heatmap was carried out using MeV (v4.9.0) software [15]. Below: Pathway maps indicating the expression of its constituent metabolites. G1P, glucose 1 phosphate; G6P, glucose 6 phosphate; F6P, fructose 6 phosphate; F1,6P, fructose 1,6 phosphate; DHAP, dihydroxyacetone phosphate; 3-PG, 3-phosphoglycerate; G3P, glyceraldehyde 3 phosphate; 2-PG, 2-phosphoglycerate; PEP, phosphoenol pyruvate; 3-HBA, 3-hydroxybutyrate; b-Ala, beta alanine; BTL, betaine aldehyde; DMG, *N*,*N*-Dimethylglycine.

A heat map analysis was carried out to compare the metabolite synthesis across stages and study specific metabolites. The egg stage showed abundance of nucleotides, organic acids and phosphates involved in glycolysis pathway of the central carbon metabolism (Figure 2). Also, high amounts of glyoxylate pathway intermediates including citric acid, cis-aconitate, fumarate and malate were found in the egg stage (Figure 2). Glyoxylate pathway enzymes have been previously reported in J2 juveniles of *M. incognita* [13].Though we could detect glyoxylate cycle intermediates, we did not detect glyoxylic acid during any stage of the life cycle (Table 2). Glucose-1-phosphate, a product of glycogenolysis was found to be most abundant in the egg stage followed by J2 and female stages. It has been reported that glycogen is the most common carbohydrate reserve in nematodes and contributes to 3–20% of their dry mass [14]. Thus it can be understood that increased amounts of glycogenolysis associated metabolites at egg and J2 stage indicates active carbon metabolism and use up of reserve carbon sources. Therefore, we understand that during the egg and the J2 stage, several pathways of the central carbon metabolism are activated.

**Figure 3:**
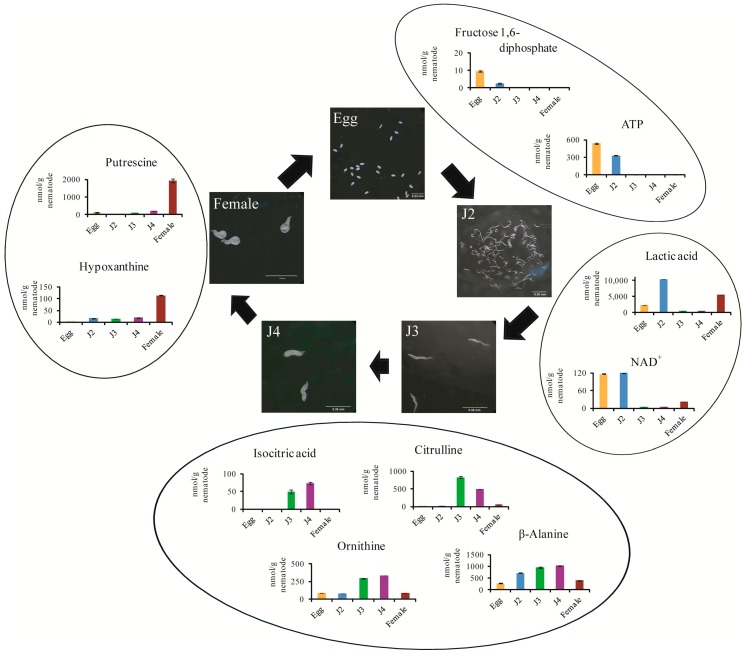
Morphology of the root-knot nematode *Meloidogyne incognita* at different stages of development and stage-specific highly expressed metabolites at each stage of the life cycle; ATP, adenosine triphosphate; NAD^+^, nicotinamide adenine dinucleotide. All metabolites concentrations were calculated by normalizing the peak area of each metabolite samples with respect to the area of the internal standard and by using standard curves, which were obtained by single-point (100 µM) calibrations. Scale bars in the representative stage images are egg (0.2 mm), J2 (0.4 mm), J3 (0.4 mm), J4 (0.4 mm) and female (1.0 mm).

Metabolites highly expressed in J2 stage also formed a group containing cGMP, AMP, IMP, lactic acid, creatine, GABA, and glycine (Figure 1B). The Cori cycle product, lactic acid, is produced in muscles during intense activity. So the highly motile nature of J2 stage supports the presence of high amounts of lactic acid during this stage (Figure 3). The basic structural protein collagen has been reported to be primarily composed of proline, hydroxyproline, and glycine [16]. High amounts of glycine and hydroxyproline in J2 suggest key muscle development. Neurotransmitter GABA which regulates muscle tone was also present in very high concentrations in J2 stage (Figure 2). GABA has already been reported to be present during J2 stage of *M. incognita* [17]. In our studies, high levels of oxidized glutathione were observed in the egg and J2 stages compared to J3, J4, and females. The metabolite glutathione (GSH) in its oxidized and reduced forms has been found to be essential for nematode infection of the plant as it regulates the metabolic activity in giant cells [18]. Therefore, we can also hypothesize that the high levels of GSH observed in the egg and J2 stages may have a role in the formation of giant cells upon infection.

Metabolites homoserine, creatinine, ornithine, sarcosine, β-alanine, isocitric acid, ribose-5-phosphate, and betaine aldehyde were found to be highly expressed in the J3 and J4 stages (Figure 2). The amino acid homoserine has been reported to act as an intermediate molecule in the synthesis of essential amino acids methionine, threonine, and isoleucine [19]. Isocitrate and betaine aldehyde are also part of the amino acid metabolism in cells whereas creatinine and ornithine have function in the urea cycle. Sarcosine and β-alanine are produced in the degradation pathways of amino acids, proteins and nucleotides. Citrulline, specifically high in the J3 stage, is also an intermediate in the urea cycle (Figure 3). The female stage also showed high levels of collagen precursors proline, hydroxyproline and glycine which can be due to egg production in the adult stage (Figure 2). Moreover, choline which is a major element of cell membrane was also found to be highly synthesized in the female stage [14]. Other major metabolites included putrescine, spermidine, reduced glutathione, *S*-adenosylmethionine, nucleobases, 2-oxoglutaric acid, gluconate, pyruvate, and succinate (Figure 2 and Figure 3). The metabolite 2-oxoglutaric acid has recently been reported to functional in extending lifespan in nematodes [20] and in our data we found 2-oxoglutaric acid to be present in very high quantities during the female stage (Table 3).

## 3. Materials and Methods

### 3.1. Sample Preparation

The root-knot nematode *Meloidogyne incognita* is constantly maintained in our laboratory in tomato plants (*Solanum lycopersicum* var. Rutgers) in a greenhouse maintained at 25 °C. Eggs from the plant roots were collected by washing the roots, then they were cleaned and treated with 10% NaClO for 5 min. The wash solution was passed through a 25 µM mesh to trap the eggs which were used to infect new tomato plants (~1000 eggs/plant). The plants were continuously monitored to check the stage of the nematodes and quickly collected at the required stages. Sample collection was initiated at six weeks after infection when J3 stage was identified in the roots by manual inspection using a stereo microscope. To collect stages J3, J4, and female, infected roots were washed, chopped, and treated with 7.7% cellulase and 15.4% pectinase followed by washing and filtering through a 75 µM mesh. A combination of 15 mL of cellulose and 30 mL of pectinase was used for five roots. The samples retained on the filter were re-dissolved in water and nematodes were handpicked using a pipette under a stereo microscope. Eggs were collected by sucrose gradient centrifugation (35% sucrose, 1500 rpm, room temperature) after hypochlorite treatment (10% NaClO) of washed roots and J2 samples were obtained by hatching of the eggs at 25 °C for five days in double distilled autoclaved water and filtering using 5–7 KIMTECH ScienceWipers^®^ (Yuhan-Kimberly Professional, Seoul, Korea) on Petri dish placed on a lab table. Samples were collected from plants grown in four consecutive seasons to obtain an adequate amount of samples for analyses.

Two hundred milligrams (200 mg) of samples for each stage at egg, J2, J3, J4, and female were collected from the roots of tomato plants and were snap frozen using liquid nitrogen followed by storage at −80 °C until further processing. For metabolite extraction, 50 mg of each samples were taken in a 2.0 mL cryotube containing Zirconia beads (5mm Ø × 1, 3mm Ø × 5) and 500 µL of methanol was added. The cryotubes were fixed to a beads-shocker and homogenized at 4000 rpm, for 60 s (at 4 °C) twice. Then 500 µL of chloroform and 200 µL of MilliQ water were added to each tube and vortexed for 30 s. The tubes were then centrifuged at 2300× *g* for 5 min (at 4 °C) and the upper aqueous layer was carefully removed (~400 µL) and added to a pre-washed (using 250 µL MilliQ water) microcentrifugal filters and centrifuged at 9100× *g* until the solutions were completely filtered (4 to 6 h). The filtrates containing the metabolites were dried under vacuum. The dried samples were stored and transported at −80°C and re-suspended in ultrapure water immediately before the measurement. The samples were divided into three parts for CE-TOF/MS analysis (Table 4).

### 3.2. Measurement

Metabolite quantification was carried out at Human Metabolome Technologies (HMT) Japan using a CE-TOF/MS (Capillary electrophoresis-Time of Flight Mass Spectrometer) system (Agilent Technologies Inc. Santa Clara, CA, USA) in anion and cation modes followed by extraction of the peaks using automatic integration software (MasterHands ver. 2.13.0.8.h developed at Keio University). The compounds were analyzed with the following conditions. CE-TOFMS was carried out using an Agilent CE Capillary Electrophoresis System equipped with an Agilent 6210 Time of Flight mass spectrometer, Agilent 1100 isocratic HPLC pump, Agilent G1603A CE-MS adapter kit, and Agilent G1607A CE-ESI-MS sprayer kit (Agilent Technologies, Waldbronn, Germany). The system was controlled by Agilent G2201AA ChemStation software version B.03.01 for CE (Agilent Technologies). Fused silica capillary (50 μm i.d × 80 cm total length) was used and the run buffer included cation buffer solution (*p*/*n*: H3301-1001) and anion run buffer (*p*/*n*: I3302-1023) which were also used for rinsing. Electrospray ionization-mass spectrometry (ESI-MS) was conducted both in positive and negative ion modes and sample injection parameters included pressure injection 50 mbar for 10 s, CE voltage of 27 kV (cation) 30 kV (anion), MS capillary voltage of 4000 V (cation), 3500 (anion), and scan range of *m*/*z* 50–1000. The samples were diluted two-fold and five-fold for the measurement of cation and anion modes, respectively, to improve analysis qualities in the cation mode of the CE-MS analysis [21,22].

### 3.3. Data Processing and Analysis

Peaks detected in CE-TOFMS analysis were extracted using automatic integration software (MasterHands ver. 2.13.0.8.h developed at Keio University) in order to obtain peak information including *m*/*z*, migration time (MT), and peak area. Signal peaks corresponding to isotopomers, adduct ions, and other product ions of known metabolites were excluded, and all signal peaks potentially corresponding to authentic compounds were extracted, and then their migration time (MT) was normalized using those of the internal standards. Thereafter, the alignment of peaks was performed according to the *m*/*z* values and normalized MT values. Finally, peak areas were normalized against those of the internal standards, MetSul (methionine sulfone) and CSA (D-camphor-10-sulfonic acid) for cations and anions, respectively. The resultant relative area values were further normalized by sample amount. Annotation tables were produced from CE-ESI-TOFMS measurement of standard compounds, and were aligned with the datasets according to similar m/z values and normalized MT values. The peak to relative peak area was calculated by the following equation. The peak detection limit was determined based on signal-noise ration; *S*/*N* = 3.

$$\mathrm{Relative}\;\mathrm{Peak}\;\mathrm{Area}\;=\frac{\;\mathrm{Metabolite}\;\mathrm{Peak}\;\mathrm{Area}}{\mathrm{Internal}\;\mathrm{Standard}\;\mathrm{Peak}\;\mathrm{Area}\;\times \;\mathrm{Sample}\;\mathrm{Amount}}$$

### 3.4. Quantitative Estimation of Metabolites and Statistical Analyses

Absolute quantification was performed in 110 metabolites including glycolytic and TCA cycle intermediates, amino acids, and nucleic acids. All the metabolite concentrations were calculated by normalizing the peak area of each metabolite with respect to the area of the internal standard and by using standard curves, which were obtained by single-point (100 µM) calibrations. Principal component analysis (PCA) was performed by SIMCA v13.0.3. Quantitative expression of metabolites was normalized using PeakStat ver 3.18 (HMT, Tsuruoka, Japan) and heat maps generated using MeV software v4.9.0 [15]

## 4. Conclusions

Overall, we observe active energy metabolism to take place during egg stage; growth and motility related metabolites in J2; amino acid metabolism and urea cycle related compounds in J3 and J4 followed by cellular homeostasis and new nucleic acids, cell synthesis related metabolites in females. Specific metabolites can now be selected as biomarkers and used to study their physiological roles in the development of the root-knot nematode *Meloidogyne incognita*.

## Figures and Tables

**Table 1:** Primary metabolic pathways occurring in *M. incognita* with their constituting groups of metabolites.

Metabolism	Pathways and Groups	Metabolites Involved
Amino acid metabolism	Bile acids, methylation cycle, sulfur amino acids	2-Hydroxybutyric acid, Cys, Gly, Glycolic acid, Glyoxylic acid, Lys, Met, *S*-Adenosylmethionine, Ser, Thr
BCAA and aromatic amino acids metabolism	Aromatic amino acids, branched chain amino acids	2-Oxoisovaleric acid, Ala, Anthranilic acid, Ile, Leu, Phe, Trp, Tyr, Tyramine, Val
Central carbon metabolism	Glycolysis/gluconeogenesis, nucleotide sugar/amino sugar, pentose phosphate pathway, tricarboxylic acid (TCA) cycle	2-Oxoglutaric acid, 2-Phosphoglyceric acid, 3-Hydroxybutyric acid, 3-Phosphoglyceric acid, 6-Phosphogluconic acid, ADP, Ala, Asp, Arg, ATP, Acetyl CoA_divalent, cis-Aconitic acid, Citric acid, CoA_divalent, Dihydroxyacetone phosphate, Erythrose 4-phosphate, Fructose 6-phosphate, Fructose 1,6-diphosphate, Fumaric acid, Glu, Glucose 1-phosphate, Glucose 6-phosphate, Glyceraldehyde 3-phosphate, Glycerol 3-phosphate, Isocitric acid, Lactic acid, Malic acid, Malonyl CoA_divalent, NAD+, NADP+, Phosphoenolpyruvic acid, PRPP, Pyruvic acid, Ribose 5-phosphate, Ribulose 5-phosphate, Sedoheptulose 7-phosphate, Succinic acid
Lipid metabolism	Carnitine, choline metabolism	3-Hydroxybutyric acid, 3-Phosphoglyceric acid, Acetyl CoA_divalent, Betaine, Betaine aldehyde, Choline, Dihydroxyacetone phosphate, Glyceraldehyde 3-phosphate, Glycerol 3-phosphate, Malonyl CoA_divalent, N,N-Dimethylglycine, Pyruvic acid, Sarcosine
Metabolism of coenzymes	Biotin, folate, nicotinamide, riboflavin, Vitamin B6, C	β-Ala, Acetyl CoA_divalent, CoA_divalent, CTP, Cys, NAD^+^, NADP^+^, Ribose 5-phosphate
Nucleotide metabolism	Purine and pyrimidine synthseis	β-Ala, ADP, Adenine, Adenosine, AMP, Asp, ATP, cAMP, CDP, cGMP, CMP, CTP, Cytidine, Cytosine, dATP, dCTP, dTDP, dTMP, dTTP, GDP, GMP, GTP, Guanine, Guanosine, Hypoxanthine, IMP, Inosine, PRPP, Thymidine, Thymine, UDP, UMP, Uracil, Uridine, UTP
Urea cycle-related metabolism	Creatine metabolism, glutathione metabolism, urea cycle, polyamines	2-Oxoglutaric acid, β-Ala, Ala, Arg, Asp, Asn, Carnosine, Citrulline, Creatine, Creatinine, Cys, Fumaric acid, GABA, Gln, Glu, Glutathione (GSH), Glutathione (GSSG)_divalent, Gly, His, Hydroxyproline, Lactic acid, Malic acid, Ornithine, Pro, Putrescine, Pyruvic acid, Spermidine, Succinic acid, Spermine
Miscellaneous metabolism	–	Gluconic acid, Homoserine

AMP, adenosine monophosphate; ADP, adenosine diphosphate; ATP, adenosine triphosphate; Ala, alanine; Asn, asparagine; Arg, arginine; Asp, aspartic acid; β-Ala, β alanine; cAMP, cyclic adenosine monophosphate; cGMP, cyclic guanosine monophosphate; CDP, cytidine diphosphophate; CTP, cytidine triphosphophate; Cys, cysteine; DHAP, dihydroxyacetone phosphate; dTMP, deoxythymidine monophosphate; dTDP, deoxythymidine diphosphate; GABA, gamma-aminobutyric acid; Glu, glutamic acid; Gln, glutamine; GMP, guanosine monophosphate; GDP, guanosine diphosphate; GTP, guanosine triphosphate; IMP, inosine monophosphate; Lys, lysine; Met, methionine; NAD^+^, nicotinamide adenine dinucleotide; NADP^+^, nicotinamide adenine dinucleotide phosphate; Pro, proline; PRPP, phosphoribosyl pyrophosphate; Trp, tryptophan; UMP, uridine monophosphate; UDP, uridine diphosphate; UTP, uridine triphosphate.

**Table 2:** Quantitative estimation of metabolites at various stages of the root-knot nematode life cycle.

Mode	CAS Number	KEGG ID	HMDB ID	Metabolite	Concentration (nmol/g)									
					Egg		J2		J3		J4		Female	
					Mean	S.D.	Mean	S.D.	Mean	S.D.	Mean	S.D.	Mean	S.D.
Anion	600-15-7	C05984	HMDB00008	2-Hydroxybutyric acid	1.07	0.06	5.06	0.20	N.D.	N.D.	N.D.	N.D.	2.09	0.16
Anion	64-15-3	C00026	HMDB00208	2-Oxoglutaric acid	45.14	1.39	N.D.	N.D.	23.30	1.98	41.43	2.18	222.14	3.25
Anion	759-05-7	C00141	HMDB00019	2-Oxoisovaleric acid	N.D.	N.D.	N.D.	N.D.	N.D.	N.D.	N.D.	N.D.	N.D.	N.D.
Anion	2553-59-5	C00631	HMDB03391	2-Phosphoglyceric acid	N.D.	N.D.	N.D.	N.D.	N.D.	N.D.	N.D.	N.D.	N.D.	N.D.
Anion	300-85-6	C01089, C03197	HMDB00011, HMDB00357, HMDB00442	3-Hydroxybutyric acid	88.12	1.24	34.20	1.89	10.33	0.56	4.68	0.27	15.02	0.49
Anion	820-11-1	C00197	HMDB00807	3-Phosphoglyceric acid	7.91	0.53	20.77	1.17	N.D.	N.D.	N.D.	N.D.	18.11	1.02
Anion	921-62-0	C00345	HMDB01316	6-Phosphogluconic acid	12.69	0.63	N.D.	N.D.	N.D.	N.D.	N.D.	N.D.	N.D.	N.D.
Anion	72-89-9	C00024	HMDB01206	Acetyl CoA divalent	N.D.	N.D.	N.D.	N.D.	N.D.	N.D.	N.D.	N.D.	N.D.	N.D.
Anion	58-64-0	C00008	HMDB01341	ADP	554.88	8.82	506.60	11.22	14.97	1.15	16.34	0.75	65.44	1.51
Anion	61-19-8	C00020	HMDB00045	AMP	366.11	4.88	1737.14	19.64	38.69	1.40	33.98	0.14	416.29	8.59
Anion	56-65-5	C00002	HMDB00538	ATP	537.74	10.90	331.66	6.80	2.19	0.02	6.32	0.05	3.06	0.12
Anion	60-92-4	C00575	HMDB00058	cAMP	1.85	0.22	1.31	0.09	0.69	0.16	0.88	0.09	1.51	0.09
Anion	63-38-7	C00112	HMDB01546	CDP	8.99	0.06	1.49	0.14	N.D.	N.D.	N.D.	N.D.	1.43	0.02
Anion	7665-99-8	C00942	HMDB01314	cGMP	N.D.	N.D.	3.39	0.28	N.D.	N.D.	N.D.	N.D.	N.D.	N.D.
Anion	585-84-2	C00417	HMDB00072	*cis*-Aconitic acid	18.05	0.58	4.36	0.41	N.D.	N.D.	N.D.	N.D.	7.61	0.47
Anion	77-92-9	C00158	HMDB00094	Citric acid	863.04	4.62	203.15	0.93	N.D.	N.D.	N.D.	N.D.	739.20	19.60
Anion	63-37-6	C00055	HMDB00095	CMP	43.37	0.79	13.38	0.67	3.17	0.14	3.42	0.11	31.10	0.98
Anion	85-61-0	C00010	HMDB01423	CoA_divalent	N.D.	N.D.	N.D.	N.D.	N.D.	N.D.	N.D.	N.D.	N.D.	N.D.
Anion	65-47-4	C00063	HMDB00082	CTP	6.00	0.30	N.D.	N.D.	N.D.	N.D.	N.D.	N.D.	N.D.	N.D.
Anion	1927-31-7	C00131	HMDB01532	dATP	N.D.	N.D.	N.D.	N.D.	N.D.	N.D.	N.D.	N.D.	N.D.	N.D.
Anion	2056-98-6	C00458	HMDB00998	dCTP	N.D.	N.D.	N.D.	N.D.	N.D.	N.D.	N.D.	N.D.	N.D.	N.D.
Anion	57-04-5	C00111	HMDB01473	Dihydroxyacetone phosphate	2.75	0.15	N.D.	N.D.	N.D.	N.D.	N.D.	N.D.	N.D.	N.D.
Anion	491-97-4	C00363	HMDB01274	dTDP	1.37	0.19	N.D.	N.D.	N.D.	N.D.	N.D.	N.D.	N.D.	N.D.
Anion	365-07-1	C00364	HMDB01227	dTMP	14.34	0.22	N.D.	N.D.	N.D.	N.D.	N.D.	N.D.	0.86	N.D.
Anion	365-08-2	C00459	HMDB01342	dTTP	N.D.	N.D.	N.D.	N.D.	N.D.	N.D.	N.D.	N.D.	N.D.	N.D.
Anion	585-18-2	C00279, C03604	HMDB01321	Erythrose 4-phosphate	N.D.	N.D.	N.D.	N.D.	N.D.	N.D.	N.D.	N.D.	N.D.	N.D.
Anion	488-69-7	C00354	HMDB01058	Fructose 1,6-diphosphate	9.63	0.41	2.43	0.34	N.D.	N.D.	N.D.	N.D.	N.D.	N.D.
Anion	643-13-0	C05345, C00085	HMDB00124	Fructose 6-phosphate	46.38	2.14	20.55	1.01	2.12	0.33	2.92	0.24	16.58	1.01
Anion	110-17-8	C00122	HMDB00134	Fumaric acid	167.88	1.72	91.56	0.88	10.52	1.16	12.76	0.52	98.77	1.04
Anion	146-91-8	C00035	HMDB01201	GDP	305.03	1.56	89.87	0.37	2.02	0.06	4.81	0.31	9.30	0.21
Anion	526-95-4	C00257	HMDB00625	Gluconic acid	50.80	0.25	32.47	1.28	7.66	0.79	13.12	0.44	83.21	1.58
Anion	59-56-3	C00103	HMDB01586	Glucose 1-phosphate	51.11	0.97	31.64	2.26	1.68	0.20	3.00	0.31	18.70	0.18
Anion	56-73-5	C00668, C01172, C00092	HMDB01401	Glucose 6-phosphate	267.74	3.48	136.92	0.24	8.83	1.14	14.71	0.15	70.88	1.69
Anion	142-10-9	C00118, C00661	HMDB01112	Glyceraldehyde 3-phosphate	N.D.	N.D.	N.D.	N.D.	N.D.	N.D.	N.D.	N.D.	N.D.	N.D.
Anion	57-03-4	C00093	HMDB00126	Glycerol 3-phosphate	621.13	8.99	424.51	3.17	29.70	1.48	42.14	0.87	243.07	3.39
Anion	79-14-1	C00160	HMDB00115	Glycolic acid	N.D.	N.D.	N.D.	N.D.	N.D.	N.D.	N.D.	N.D.	N.D.	N.D.
Anion	298-12-4	C00048	HMDB00119	Glyoxylic acid	N.D.	N.D.	N.D.	N.D.	N.D.	N.D.	N.D.	N.D.	N.D.	N.D.
Anion	85-32-5	C00144	HMDB01397	GMP	2955.59	31.13	672.17	8.93	37.86	3.13	65.08	0.31	710.46	3.40
Anion	86-01-1	C00044	HMDB01273	GTP	250.09	1.98	115.69	0.56	0.69	0.09	3.29	0.12	N.D.	N.D.
Anion	131-99-7	C00130	HMDB00175	IMP	12.55	0.27	54.96	2.15	2.31	0.04	2.18	0.15	17.54	0.84
Anion	320-77-4	C00311	HMDB00193	Isocitric acid	N.D.	N.D.	N.D.	N.D.	49.78	5.39	73.33	3.67	N.D.	N.D.
Anion	79-33-4	C00186, C00256, C01432	HMDB00190, HMDB01311	Lactic acid	2423.86	40.43	10,324.40	111.33	428.02	34.65	509.05	7.74	5550.06	52.39
Anion	6915-15-7	C00149, C00497, C00711	HMDB00156, HMDB00744	Malic acid	1160.34	6.70	330.59	3.49	50.39	4.78	62.72	2.36	618.25	10.98
Anion	524-14-1	C00083	HMDB01175	Malonyl CoA divalent	N.D.	N.D.	N.D.	N.D.	N.D.	N.D.	N.D.	N.D.	N.D.	N.D.
Anion	53-84-9	C00003	HMDB00902	NAD^+^	116.73	0.98	119.54	1.24	4.72	0.45	5.14	0.46	22.35	0.51
Anion	53-59-8	C00006	HMDB00217	NADP^+^	10.27	0.28	8.95	0.34	0.71	0.14	0.98	0.13	6.03	0.55
Anion	138-08-9	C00074	HMDB00263	Phosphoenolpyruvic acid	N.D.	N.D.	N.D.	N.D.	N.D.	N.D.	N.D.	N.D.	N.D.	N.D.
Anion	7540-64-9	C00119	HMDB00280	PRPP	N.D.	N.D.	N.D.	N.D.	N.D.	N.D.	N.D.	N.D.	N.D.	N.D.
Anion	127-17-3	C00022	HMDB00243	Pyruvic acid	36.13	0.94	N.D.	N.D.	N.D.	N.D.	N.D.	N.D.	66.01	2.17
Anion	3615-55-2	C00117	HMDB01548	Ribose 5-phosphate	N.D.	N.D.	N.D.	N.D.	1.37	0.12	1.80	0.05	N.D.	N.D.
Anion	4151-19-3	C00199, C01101	HMDB00618	Ribulose 5-phosphate	8.30	0.24	8.97	0.22	N.D.	N.D.	N.D.	N.D.	14.97	0.33
Anion	2646-35-7	C05382	HMDB01068	Sedoheptulose 7-phosphate	12.13	0.33	12.92	0.51	3.25	0.21	3.32	0.44	15.64	0.13
Anion	110-15-6	C00042	HMDB00254	Succinic acid	389.50	1.59	740.35	9.90	69.36	6.18	77.51	2.79	965.41	12.70
Anion	58-98-0	C00015	HMDB00295	UDP	9.55	0.16	12.54	0.21	0.52	0.03	1.40	0.08	1.47	N.D.
Anion	58-97-9	C00105	HMDB00288	UMP	325.43	7.71	132.78	0.76	10.88	0.69	11.62	0.57	55.75	0.58
Anion	63-39-8	C00075	HMDB00285	UTP	4.32	0.26	10.49	0.59	N.D.	N.D.	N.D.	N.D.	N.D.	N.D.
Cation	73-24-5	C00147	HMDB00034	Adenine	4.74	0.18	10.92	0.11	69.38	3.08	3.20	0.17	158.47	8.28
Cation	58-61-7	C00212	HMDB00050	Adenosine	323.29	7.26	283.32	3.52	52.43	0.67	29.05	1.01	725.67	10.05
Cation	56-41-7	C00041, C00133, C01401	HMDB00161, HMDB01310	Ala	16,280.23	224.20	14,327.99	372.62	1.06	0.37	1.23	0.19	6090.10	125.55
Cation	118-92-3	C00108	HMDB01123	Anthranilic acid	0.64	N.D.	3.04	0.20	2.51	0.21	3.65	0.22	4.70	0.27
Cation	74-79-3	C00062, C00792	HMDB00517, HMDB03416	Arg	10,330.56	33.44	15,549.23	73.92	51.97	0.77	111.63	1.64	6947.89	320.07
Cation	70-47-3	C00152, C01905, C16438	HMDB00168	Asn	20556.17	955.77	5769.99	83.27	69.57	0.56	174.37	2.54	4996.98	82.13
Cation	56-84-8	C00049, C00402, C16433	HMDB00191, HMDB06483	Asp	5673.45	63.67	498.72	30.06	39.34	0.69	34.87	0.86	134.69	1.78
Cation	107-43-7	C00719	HMDB00043	Betaine	3957.37	60.93	6797.51	86.18	577.34	5.74	645.37	6.56	3096.42	53.51
Cation	7418-61-3	C00576	HMDB01252	Betaine aldehyde	1.44	0.06	1.46	0.11	4.75	0.08	3.56	0.11	6.21	0.25
Cation	305-84-0	C00386	HMDB00033	Carnosine	N.D.	N.D.	N.D.	N.D.	N.D.	N.D.	N.D.	N.D.	N.D.	N.D.
Cation	62-49-7	C00114	HMDB00097	Choline	632.80	20.58	1354.72	83.83	527.33	29.70	240.87	3.42	2415.85	118.35
Cation	372-75-8	C00327	HMDB00904	Citrulline	5.27	0.23	15.39	0.70	826.02	27.07	499.89	3.05	62.69	1.49
Cation	57-00-1	C00300	HMDB00064	Creatine	0.46	0.11	0.82	0.13	0.23	0.02	0.37	0.06	0.75	0.15
Cation	60-27-5	C00791	HMDB00562	Creatinine	N.D.	N.D.	N.D.	N.D.	0.19	0.04	0.27	0.06	N.D.	N.D.
Cation	52-90-4	C00097, C00736, C00793	HMDB00574, HMDB03417	Cys	N.D.	N.D.	1.34	N.D.	1.41	0.69	4.63	2.61	4.51	1.45
Cation	65-46-3	C00475	HMDB00089	Cytidine	30.03	0.07	26.03	0.43	8.75	0.15	5.97	0.08	59.75	1.52
Cation	71-30-7	C00380	HMDB00630	Cytosine	6.71	0.21	1.74	0.14	0.22	0.03	0.28	0.14	N.D.	N.D.
Cation	56-12-2	C00334	HMDB00112	GABA	177.50	2.01	2303.29	23.21	288.91	5.66	384.92	1.81	1481.22	56.16
Cation	56-85-9	C00064, C00303, C00819	HMDB00641, HMDB03423	Gln	17300.56	176.06	4117.04	63.66	343.68	20.21	424.12	6.27	24,448.12	332.14
Cation	110-94-1	C00025, C00217, C00302	HMDB00148, HMDB03339	Glu	22225.19	125.32	8612.13	45.17	1797.29	6.00	1986.19	17.11	11,894.23	150.60
Cation	70-18-8	C00051	HMDB00125	Glutathione (GSH)	N.D.	N.D.	19.84	3.36	N.D.	N.D.	38.63	3.57	74.72	1.08
Cation	27025-41-8	C00127	HMDB03337	Glutathione (GSSG)_divalent	1286.46	11.50	1222.40	17.62	31.72	0.40	40.78	0.84	80.05	1.31
Cation	56-40-6	C00037	HMDB00123	Gly	3815.48	37.64	7040.48	146.29	660.89	8.12	607.23	20.22	4990.69	45.55
Cation	73-40-5	C00242	HMDB00132	Guanine	17.44	1.33	86.54	0.99	29.09	0.30	6.86	0.23	154.42	3.72
Cation	118-00-3	C00387	HMDB00133	Guanosine	5702.50	180.97	9561.62	103.37	984.23	19.26	239.66	3.80	2299.13	3.83
Cation	71-00-1	C00135, C00768, C06419	HMDB00177	His	7229.92	94.40	4581.46	30.24	431.72	10.45	396.96	12.33	3814.23	204.32
Cation	672-15-1	C00263	HMDB00719	Homoserine	160.16	1.92	102.81	1.78	407.64	10.12	439.09	35.33	123.98	1.61
Cation	51-35-4	C01157	HMDB00725	Hydroxyproline	1910.83	27.22	2513.88	23.95	48.39	0.69	85.95	1.35	618.82	16.04
Cation	68-94-0	C00262	HMDB00157	Hypoxanthine	2.03	0.10	16.92	0.92	15.50	0.33	19.17	0.49	114.16	1.07
Cation	73-32-5	C00407, C06418, C16434	HMDB00172	Ile	3033.03	122.08	1345.32	11.89	397.15	2.80	414.83	8.06	3543.29	53.03
Cation	58-63-9	C00294	HMDB00195	Inosine	1140.15	17.05	2555.87	57.42	83.80	0.71	25.21	0.10	352.40	9.11
Cation	61-90-5	C00123, C01570, C16439	HMDB00687	Leu	3868.92	16.72	1932.00	13.21	598.35	9.28	627.33	4.39	5158.57	52.56
Cation	56-87-1	C00047, C00739, C16440	HMDB00182, HMDB03405	Lys	1709.38	19.34	2481.93	82.01	2189.12	37.24	2099.76	17.56	2584.05	137.48
Cation	63-68-3	C00073, C00855, C01733	HMDB00696	Met	8.31	0.34	1716.16	7.65	322.10	5.77	296.90	5.51	2019.27	27.00
Cation	1118-68-9	C01026	HMDB00092	*N*,*N*-Dimethylglycine	N.D.	N.D.	N.D.	N.D.	N.D.	N.D.	N.D.	N.D.	N.D.	N.D.
Cation	3184-13-2	C00077, C00515, C01602	HMDB00214, HMDB03374	Ornithine	85.01	1.97	76.46	2.34	293.94	7.12	331.02	1.16	88.54	2.44
Cation	63-91-2	C00079, C02057, C02265	HMDB00159	Phe	1411.78	18.71	1200.73	8.70	251.36	0.78	260.24	1.97	1958.12	34.79
Cation	147-85-3	C00148, C00763, C16435	HMDB00162, HMDB03411	Pro	36,515.37	313.80	3676.72	16.20	922.17	3.65	1067.84	9.17	5386.30	108.09
Cation	110-60-1	C00134	HMDB01414	Putrescine	123.96	3.61	8.43	0.26	108.77	4.62	219.67	5.73	1917.93	106.44
Cation	29908-03-0	C00019	HMDB01185	*S*-Adenosylmethionine	58.17	1.08	52.85	2.48	5.41	0.33	7.13	0.21	85.41	5.95
Cation	107-97-1	C00213	HMDB00271	Sarcosine	N.D.	N.D.	6.65	2.19	39.04	0.92	57.46	0.46	3.70	0.01
Cation	56-45-1	C00065, C00716, C00740	HMDB00187, HMDB03406	Ser	5066.52	95.36	4966.82	56.61	268.66	5.61	197.29	5.93	1883.94	31.55
Cation	124-20-9	C00315	HMDB01257	Spermidine	134.24	7.48	118.12	7.12	4.44	0.45	11.56	0.54	340.81	8.75
Cation	71-44-3	C00750	HMDB01256	Spermine	N.D.	N.D.	N.D.	N.D.	N.D.	N.D.	0.53	0.60	N.D.	N.D.
Cation	72-19-5	C00188, C00820	HMDB00167	Thr	5707.51	36.45	2521.32	27.76	1.01	0.07	1.47	0.46	2756.44	17.10
Cation	50-89-5	C00214	HMDB00273	Thymidine	98.26	0.66	52.19	1.70	N.D.	N.D.	N.D.	N.D.	42.76	3.14
Cation	65-71-4	C00178	HMDB00262	Thymine	N.D.	N.D.	N.D.	N.D.	N.D.	N.D.	N.D.	N.D.	N.D.	N.D.
Cation	73-22-3	C00078, C00525, C00806	HMDB00929	Trp	300.70	4.50	3314.18	19.97	239.64	1.41	216.86	2.87	4186.06	76.92
Cation	60-18-4	C00082, C01536, C06420	HMDB00158	Tyr	10,182.96	24.33	3267.85	42.01	415.54	5.09	454.89	6.12	4772.44	69.30
Cation	51-67-2	C00483	HMDB00306	Tyramine	1.90	0.13	2.33	0.25	0.24	0.02	0.27	N.D.	2.71	0.34
Cation	66-22-8	C00106	HMDB00300	Uracil	155.80	2.87	31.52	2.33	47.56	2.84	59.05	1.37	218.25	4.27
Cation	58-96-8	C00299	HMDB00296	Uridine	238.34	0.26	131.01	2.14	53.16	0.87	38.31	1.22	566.29	4.10
Cation	72-18-4	C00183, C06417, C16436	HMDB00883	Val	3718.51	54.73	1477.94	21.13	642.70	4.18	611.43	9.42	2873.51	34.92
Cation	107-95-9	C00099	HMDB00056	β-Ala	283.93	5.28	717.11	7.15	961.43	22.47	1026.14	12.16	394.53	21.57

“Cation” and “Anion” were detected in cationic and anionic modes, respectively. N.D. (Not Detected): The target peak or metabolite was below detection limits. Ala, Choline, Ser, Pro, Val, Betaine, Thr, Leu, Ile, Asn, Asp, Gln, Glu, His, Arg, Tyr, Guanosine: Peak intensity is saturated. Relative area was computed by using ^13^C isotope ion peak.

**Table 3:** Stage-wise highly up and down-regulated metabolites of *Meloidogyne incognita.*

Action	Egg	J2	J3	J4	Female
Upregulated	3-HBA, ADP, Asp, ATP, CDP, Cytosine, GDP, Glutathione (GSSG)_divalent, NAD^+^, Pro, UDP, UMP	ADP, ATP, GDP, NAD^+^, GABA, Glutathione (GSSG)_divalent, Inosine, Met, Trp, UDP	Adenine, Citrulline	Citrulline	2-Oxoglutaric acid, Adenine, Hypoxanthine, Gln, Guanine, Met, Putrescine, Trp
Downregulated	cGMP, Citrulline, Creatinine, Cys, Hypoxanthine, Glutathione (GSH), Isocitric acid, Met, Ribose 5-phosphate, Sarcosine, Spermine	2-Oxoglutaric acid, 6-Phosphogluconic acid, CTP, Creatinine, DHAP, dTDP, dTMP, Isocitric acid, Putrescine, Pyruvic acid, Ribose 5-phosphate, Spermine	2-Hydroxybutyric acid, 3-Phosphoglyceric acid, 6-Phosphogluconic acid, Adenosine, Ala, AMP, Arg, Asn, Betaine, CDP, cGMP, cis-Aconitic acid, Citric acid, CTP, DHAP, dTDP, dTMP, Fructose 1,6-diphosphate, Fructose 6-phosphate, Fumaric acid, Gln, Glucose 1-phosphate, Glucose 6-phosphate, Glutathione (GSH), Gly, Glycerol 3-phosphate, GMP, GTP, His, Hydroxyproline, IMP, Lactic acid, Malic acid, NADP^+^, Pyruvic acid, Ribulose 5-phosphate, *S*-Adenosylmethionine, Ser, Spermidine, Spermine, Succinic acid, Thr, Thymidine, Tyr, Tyramine, UMP, UTP	2-Hydroxybutyric acid, 3-Phosphoglyceric acid, 6-Phosphogluconic acid, Adenosine, Ala, AMP, Arg, Asn, CDP, cGMP, *cis*-Aconitic acid, Citric acid, CTP, DHAP, dTDP, dTMP, Fructose 1,6-diphosphate, Fructose 6-phosphate, Fumaric acid, Gln, Glucose 1-phosphate, Gly, Glycerol 3-phosphate, GMP, GTP, Guanosine, His, Hydroxyproline, IMP, Inosine, Malic acid, NADP^+^, Pyruvic acid, Ribulose 5-phosphate, *S*-Adenosylmethionine, Ser, Spermidine, Succinic acid, Thr, Thymidine, Tyr, Tyramine, UTP	6-Phosphogluconic acid, cGMP, Creatinine, CTP, Cytosine, DHAP, dTDP, dTMP, Fructose 1,6-diphosphate, GTP, Isocitric acid, Ribose 5-phosphate, Sarcosine, Spermine, UTP

3-HBA, 3-Hydroxybutyric acid; AMP, adenosine monophosphate; ADP, adenosine diphosphate; ATP, adenosine triphosphate; Ala, alanine; Asn, asparagine; Arg, arginine; Asp, aspartic acid; β-Ala, β alanine; cAMP, cyclic adenosine monophosphate; cGMP, cyclic guanosine monophosphate; CDP, cytidine diphosphophate; CTP, cytidine triphosphophate; Cys, cysteine; DHAP, dihydroxyacetone phosphate; dTMP, deoxythymidine monophosphate; dTDP, deoxythymidine diphosphate; GABA, gamma-aminobutyric acid; Glu, glutamic acid; Gln, glutamine; GMP, guanosine monophosphate; GDP, guanosine diphosphate; GTP, guanosine triphosphate; IMP, inosine monophosphate; Lys, lysine; Met, methionine; NAD^+^, nicotinamide adenine dinucleotide; NADP^+^, nicotinamide adenine dinucleotide phosphate; Pro, proline; Trp, tryptophan; UMP, uridine monophosphate; UDP, uridine diphosphate; UTP, uridine triphosphate. Upregulation and downregulation of metabolites was determined by calculating five times over and under median fold changes respectively for all compounds.

**Table 4:** Sample details of *M. incognita* used for metabolite extraction.

Developmental Stage	Sample Run Names	Extracted Vacuum Dried Metabolite Sample (mg)
Egg	MI-E_1	3.3
	MI-E_2	3.3
	MI-E_3	3.3
J2	MI-J2_1	2.2
	MI-J2_2	2.2
	MI-J2_3	2.2
J3	MI-J3_1	6
	MI-J3_2	6
	MI-J3_3	6
J4	MI-J4_1	13
	MI-J4_2	13
	MI-J4_3	13
Female	MI-F_1	2
	MI-F_2	2
	MI-F_3	2

## Abbreviations

CE-TOF/MS	Capillary Electrophoresis-Time Of Flight/Mass Spectrometer
PPNs	Plant-Parasitic Nematodes
MI	*Meloidogyne incognita*
PCA	Principal Component Analysis
